# Supplementary material for: A Critical View of the Application of the APEX Software (Aqueous Photochemistry of Environmentally-Occurring Xenobiotics) to Predict Photoreaction Kinetics in Surface Freshwaters
Source: Molecules. 2019 Dec 18;25(1):9. doi: 10.3390/molecules25010009 (PMC7017383; doi:10.3390/molecules25010009)
Supplement: Supplementary file 1 [file molecules-25-00009-s001.zip › 3.2.4_gcc-4.4.0.zip!/3.2.4_gcc-4.4.0/3.2.4_gcc-4.4.0/doc/octave/html/interpreter/Exponents-and-Logarithms.html]

Exponents and Logarithms - Untitled


Next: Complex Arithmetic,
Up: Arithmetic


---

### 17.1 Exponents and Logarithms

— Mapping Function: **exp** (x)  
> Compute
> `e^x`
> for each element of x. To compute the matrix
> exponential, see Linear Algebra.
>
>
>
> **See also:** log.

— Mapping Function: **expm1** (x)  
> Compute
> `exp (`x`) - 1`
> accurately in the neighborhood of zero.
>
>
>
> **See also:** exp.

— Mapping Function: **log** (x)  
> Compute the natural logarithm,
> `ln (`x`)`,
> for each element of x. To compute the
> matrix logarithm, see Linear Algebra.
>
>
>
> **See also:** exp, log1p, log2, log10, logspace.

— Mapping Function: **log1p** (x)  
> Compute
> `log (1 +` x`)`
> accurately in the neighborhood of zero.
>
>
>
> **See also:** log, exp, expm1.

— Mapping Function: **log10** (x)  
> Compute the base-10 logarithm of each element of x.
>
>
>
> **See also:** log, log2, logspace, exp.

— Mapping Function: **log2** (x)  
— Mapping Function: [f, e] = **log2** (x)  
> Compute the base-2 logarithm of each element of x.
>
> If called with two output arguments, split x into
> binary mantissa and exponent so that
> `1/2 <= abs(f) < 1`
> and e is an integer. If
> `x = 0`, `f = e = 0`.
>
>
>
> **See also:** pow2, log, log10, exp.

— Function File: **nextpow2** (x)  
> If x is a scalar, return the first integer n such that
> 2^n >= abs (x).
>
> If x is a vector, return `nextpow2 (length (`x`))`.
>
>
>
> **See also:** pow2, log2.

— Function File: **nthroot** (x, n)  
> Compute the n-th root of x, returning real results for real
> components of x. For example
>
> ```
>           nthroot (-1, 3)
>           => -1
>           (-1) ^ (1 / 3)
>           => 0.50000 - 0.86603i
> ```

— Mapping Function: **pow2** (x)  
— Mapping Function: **pow2** (f, e)  
> With one argument, computes
> 2 .^ x
> for each element of x.
>
> With two arguments, returns
> f .\* (2 .^ e).
>
>
>
> **See also:** log2, nextpow2.

— Function File: **reallog** (x)  
> Return the real-valued natural logarithm of each element of x. Report
> an error if any element results in a complex return value.
>
>
>
> **See also:** log, realpow, realsqrt.

— Function File: **realpow** (x, y)  
> Compute the real-valued, element-by-element power operator. This is
> equivalent to x `.^` y, except that `realpow`
> reports an error if any return value is complex.
>
>
>
> **See also:** reallog, realsqrt.

— Function File: **realsqrt** (x)  
> Return the real-valued square root of each element of x. Report an
> error if any element results in a complex return value.
>
>
>
> **See also:** sqrt, realpow, reallog.

— Mapping Function: **sqrt** (x)  
> Compute the square root of each element of x. If x is negative,
> a complex result is returned. To compute the matrix square root, see
> Linear Algebra.
>
>
>
> **See also:** realsqrt.
